# Publisher Correction: High-entropy relaxor ferroelectric ceramics for ultrahigh energy storage

**DOI:** 10.1038/s41467-024-50284-2

**Published:** 2024-07-11

**Authors:** Haonan Peng, Tiantian Wu, Zhen Liu, Zhengqian Fu, Dong Wang, Yanshuang Hao, Fangfang Xu, Genshui Wang, Junhao Chu

**Affiliations:** 1 Key Laboratory of Inorganic Functional Materials and Devices, Shanghai Institute of Ceramics, Chinese Academy of Sciences, Shanghai, 200050 China; 2 Center of Materials Science and Optoelectronics Engineering, University of Chinese Academy of Sciences, Beijing, 100049 China; 3 State Key Laboratory of High Performance Ceramics and Superfine Microstructures, Shanghai Institute of Ceramics, Chinese Academy of Sciences, Shanghai, 200050 China; 4 Frontier Institute of Science and Technology and State Key Laboratory for Mechanical Behavior of Materials, Xi’an Jiaotong University, 710049 Xi’an, Shaanxi China; 5 State Key Laboratory of Infrared Physics, Shanghai Institute of Technical Physics, Chinese Academy of Sciences, Shanghai, 200083 China

**Keywords:** Energy storage, Ferroelectrics and multiferroics, Electronic properties and materials

Correction to: *Nature Communications* 10.1038/s41467-024-49107-1, published online 19 June 2024

The original version of this Article contained an error in the first paragraph of page 4, which incorrectly read ‘T_m,1MHz_-T_m_,_10Hz_’. The correct version states ‘T_m,1MHz_-T_m,10Hz_’ in place of ‘T_m,1MHz_-T_m_,_10Hz_’. This has been corrected in both the PDF and HTML versions of the Article.

The original version of this Article contained an error in the first paragraph of page 7, which incorrectly read ‘(S1, S1)’. The correct version states ‘(Fig. S15)’ in place of ‘(S1, S1)’. This has been corrected in both the PDF and HTML versions of the Article.

