# Supplementary material for: High-entropy relaxor ferroelectric ceramics for ultrahigh energy storage
Source: Nat Commun. 2024 Jun 19;15:5232. doi: 10.1038/s41467-024-49107-1 (PMC11187193; doi:10.1038/s41467-024-49107-1)
Supplement: Supplementary file 1 — Supplementary Information [file 41467_2024_49107_MOESM1_ESM.pdf]

High-entropy relaxor ferroelectric ceramics for ultrahigh energy storage

Supplementary Materials

Haonan Peng[1,2#], Tiantian Wu[3#], Zhen Liu[1], Zhengqian Fu[3], Dong Wang[4*], Yanshuang Hao[1], Fangfang Xu[3], Genshui Wang[1,2,3*], Junhao Chu[5]*

1, Key Laboratory of Inorganic Functional Materials and Devices, Shanghai Institute of Ceramics, Chinese Academy of Sciences, Shanghai 200050, China.

2, Center of Materials Science and Optoelectronics Engineering, University of Chinese Academy of Sciences, Beijing 100049, China.

3, State Key Laboratory of High Performance Ceramics and Superfine Microstructures, Shanghai Institute of Ceramics, Chinese Academy of Sciences, Shanghai 200050, China.

4, Frontier Institute of Science and Technology and State Key Laboratory for Mechanical Behavior of Materials, Xi'an Jiaotong University, 710049 Xi'an, Shaanxi, China.

5, State Key Laboratory of Infrared Physics, Shanghai Institute of Technical Physics, Chinese Academy of Sciences, Shanghai 200083, China.

# Haonan Peng and Tiantian Wu contributed equally to this work.

*Corresponding author: zhenliu@mail.sic.ac.cn, wang_dong1223@mail.xjtu.edu.cn, genhsuiwang@mail.sic.ac.cn.

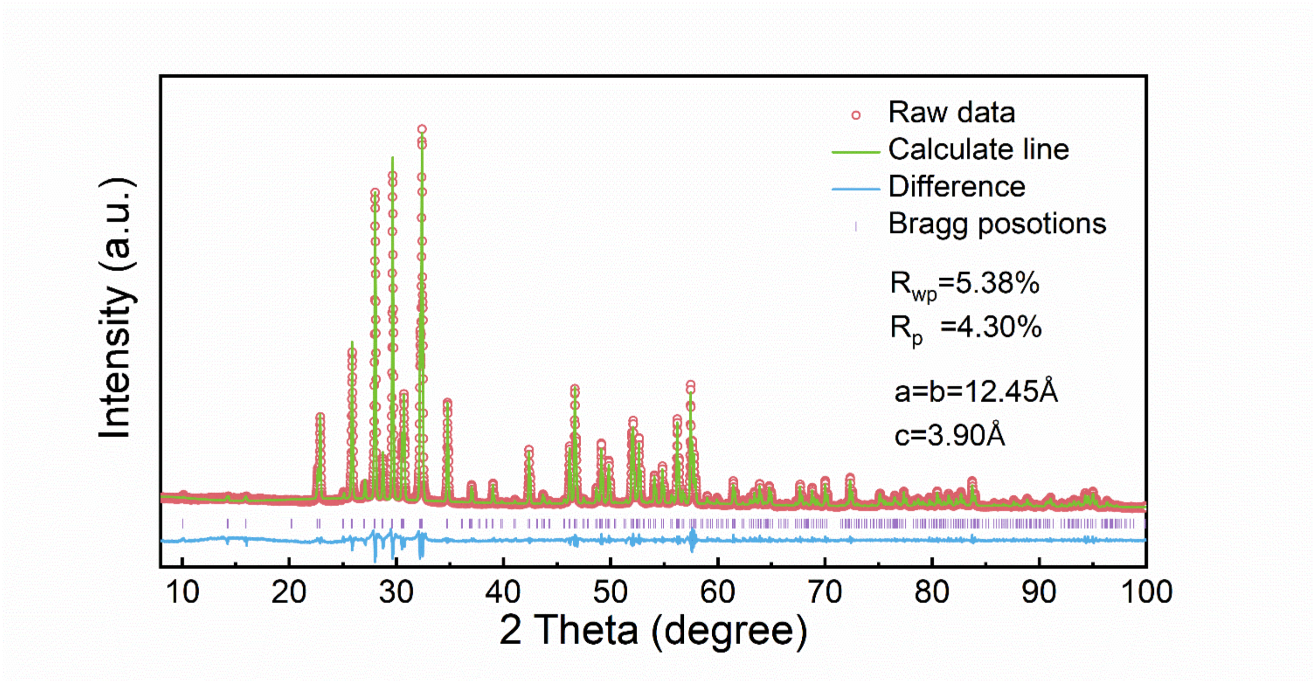

Figure S1. Rietveld refinement of XRD data of SBPLNN ceramics.

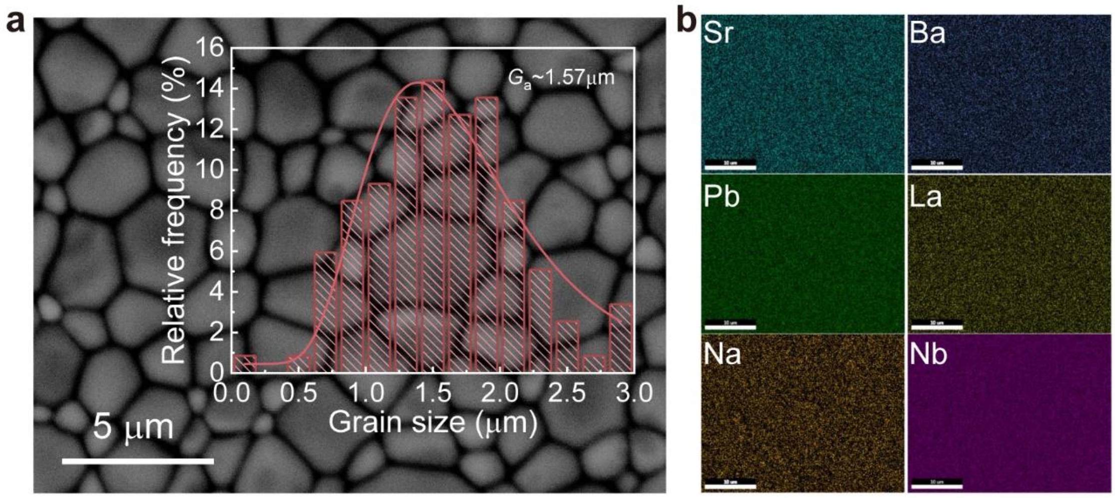

Figure S2. The SEM morphology and element distribution of SBPLNN ceramics. a SEM images and grain size distributions of SBPLNN, **b** elemental mapping of in a region of 40 × 30 μm.

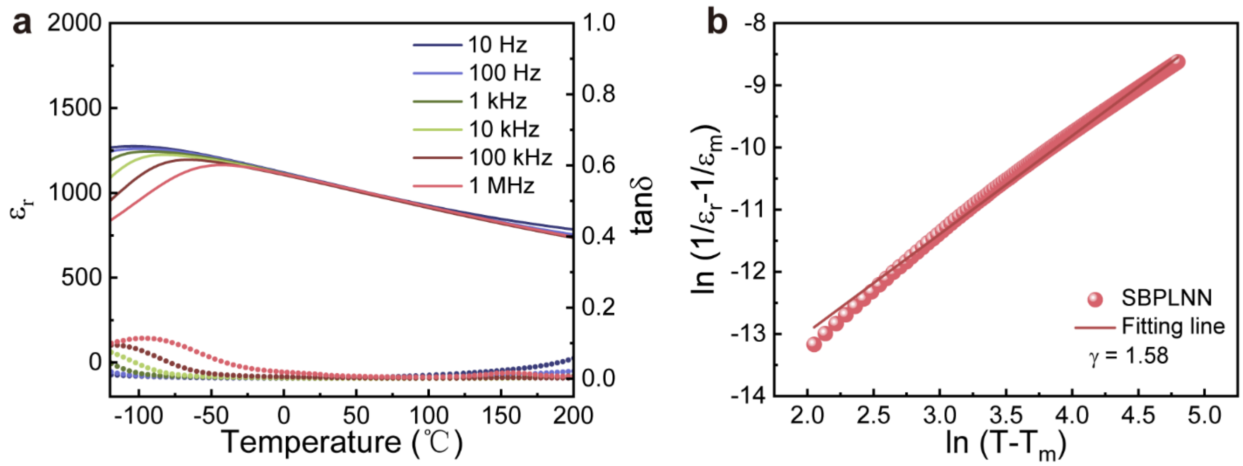

Figure S3. Dielectric properties of SBPLNN ceramics. a Temperature dependent permittivity, dielectric loss and **b** fitting of the relaxor diffuseness factor.

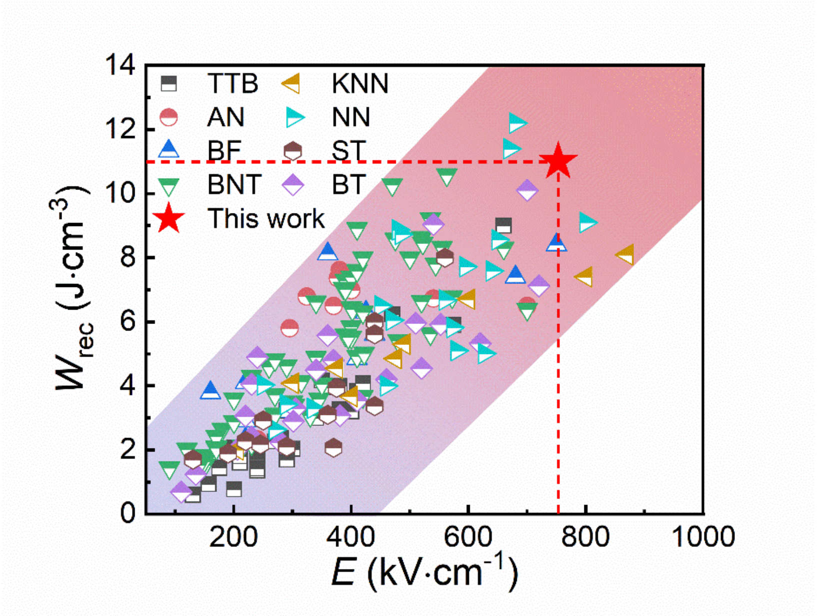

Figure S4. Comparison of the W_{rec} and E of SBPLNN ceramic with other reported tungsten bronze and perovskite structure ceramics.

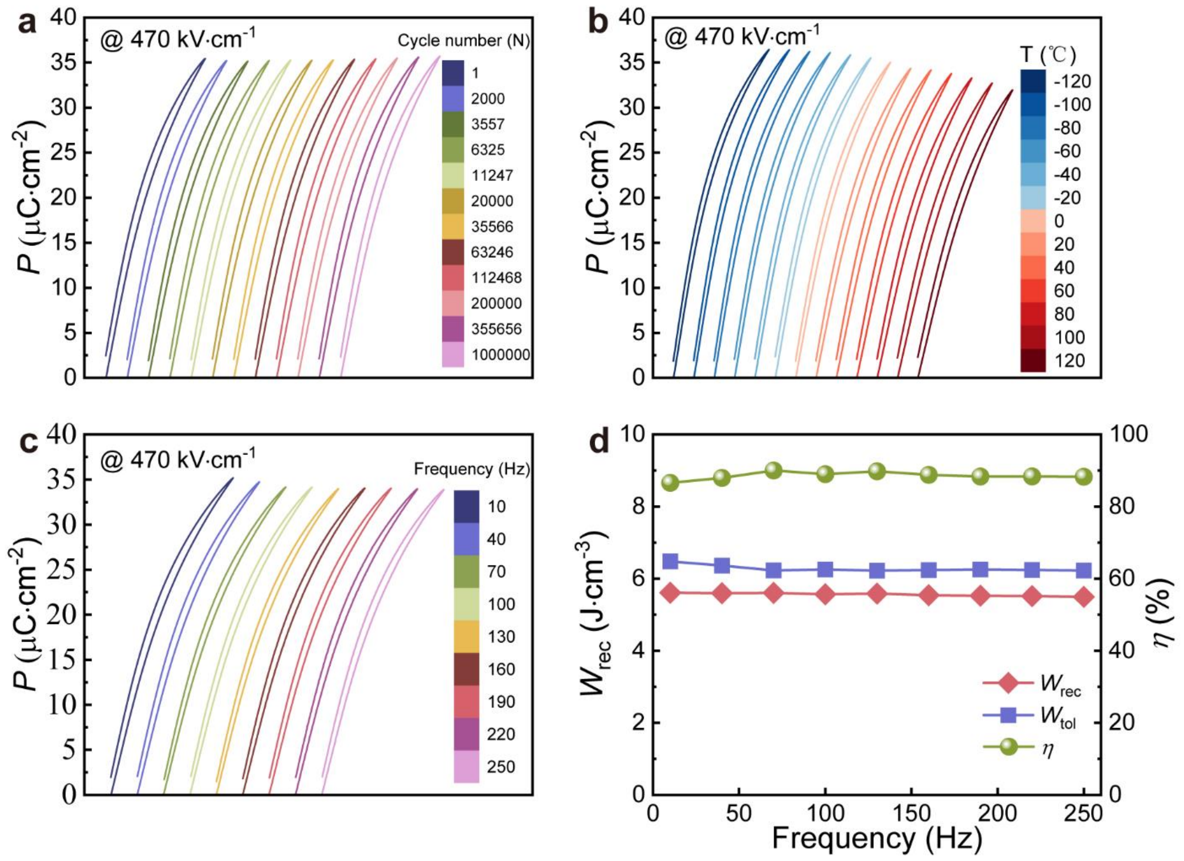

Figure S5. Stability of energy storage performance of SBPLNN ceramics. *P*-*E* loops of SBPLNN at different **a** cycle numbers, **b** temperature, and **c** frequency. **d** The energy storage performance of SBPLNN at different frequency.

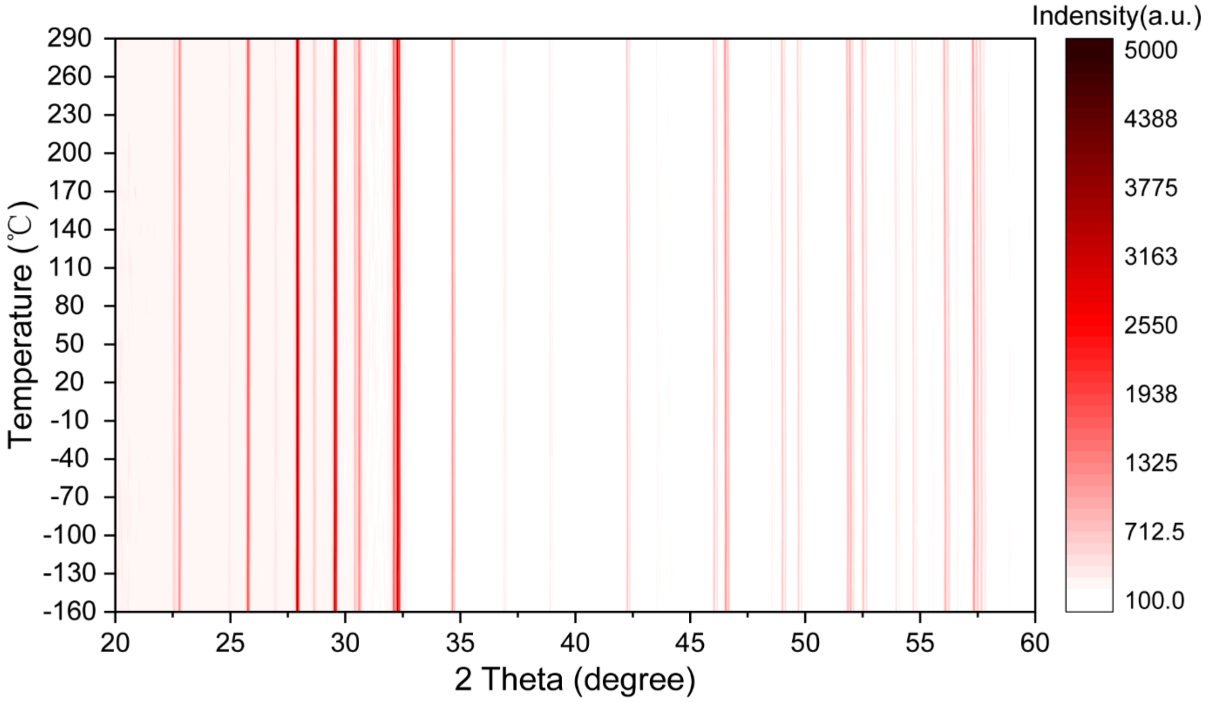

Figure S6. Temperature dependent XRD patterns of SBPLNN ceramics.

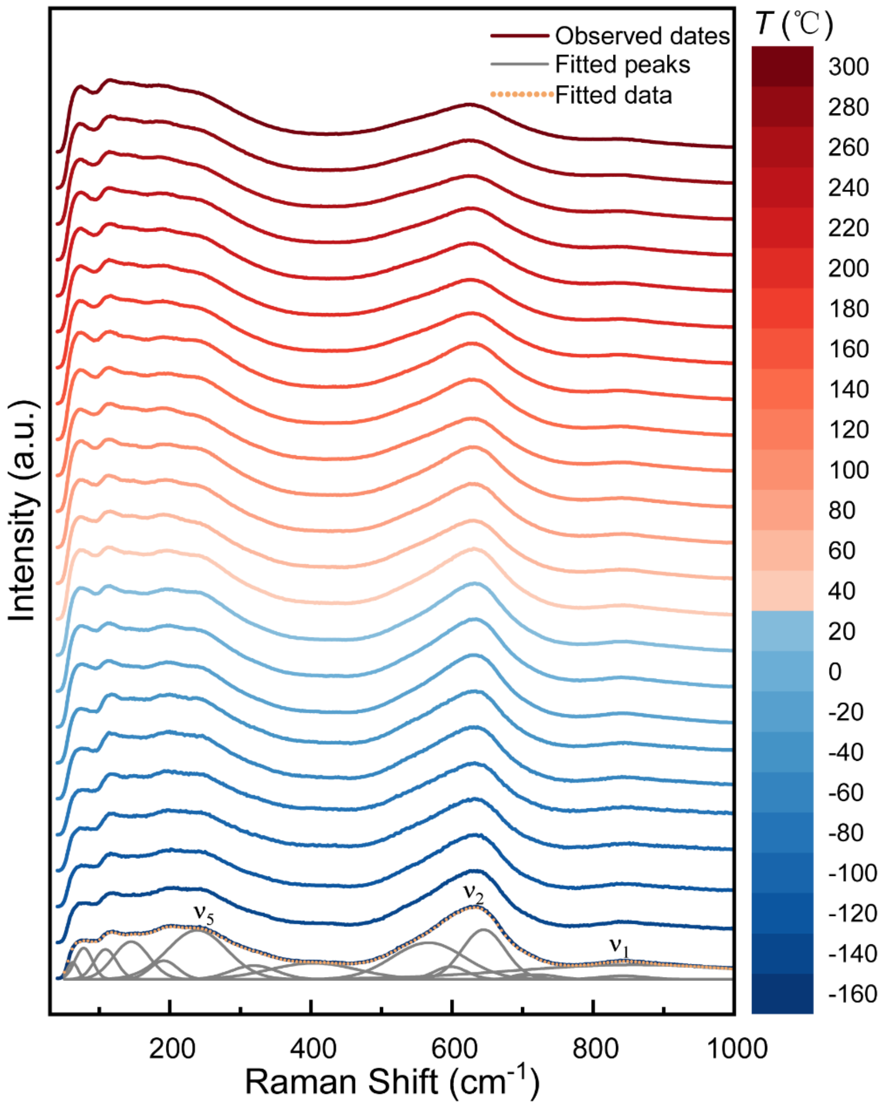

Figure S7. Temperature dependent Raman spectra of SBPLNN ceramics.

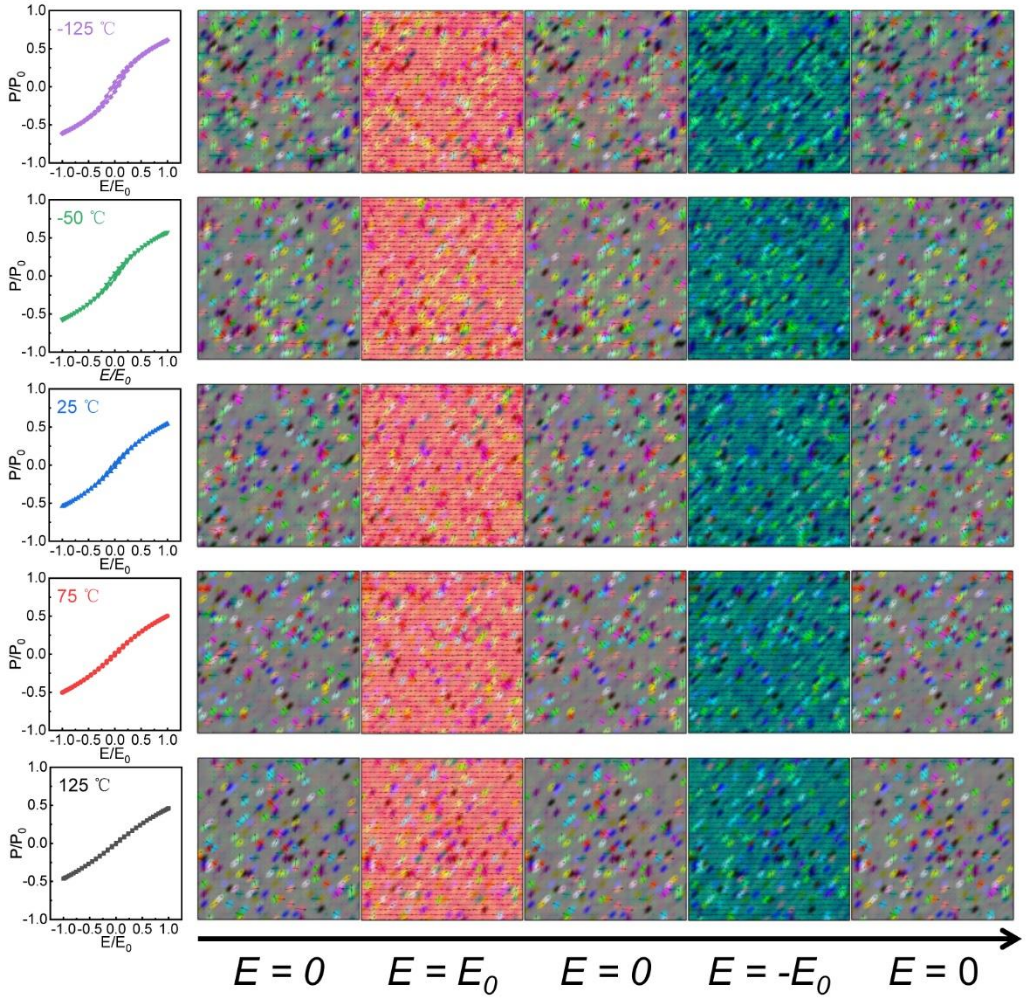

Figure S8. **Phase-field simulations results of domain structure and hysteresis loops.** Evolution of domain structure and hysteresis loops of SBPLNN ceramics under electric and temperature fields.

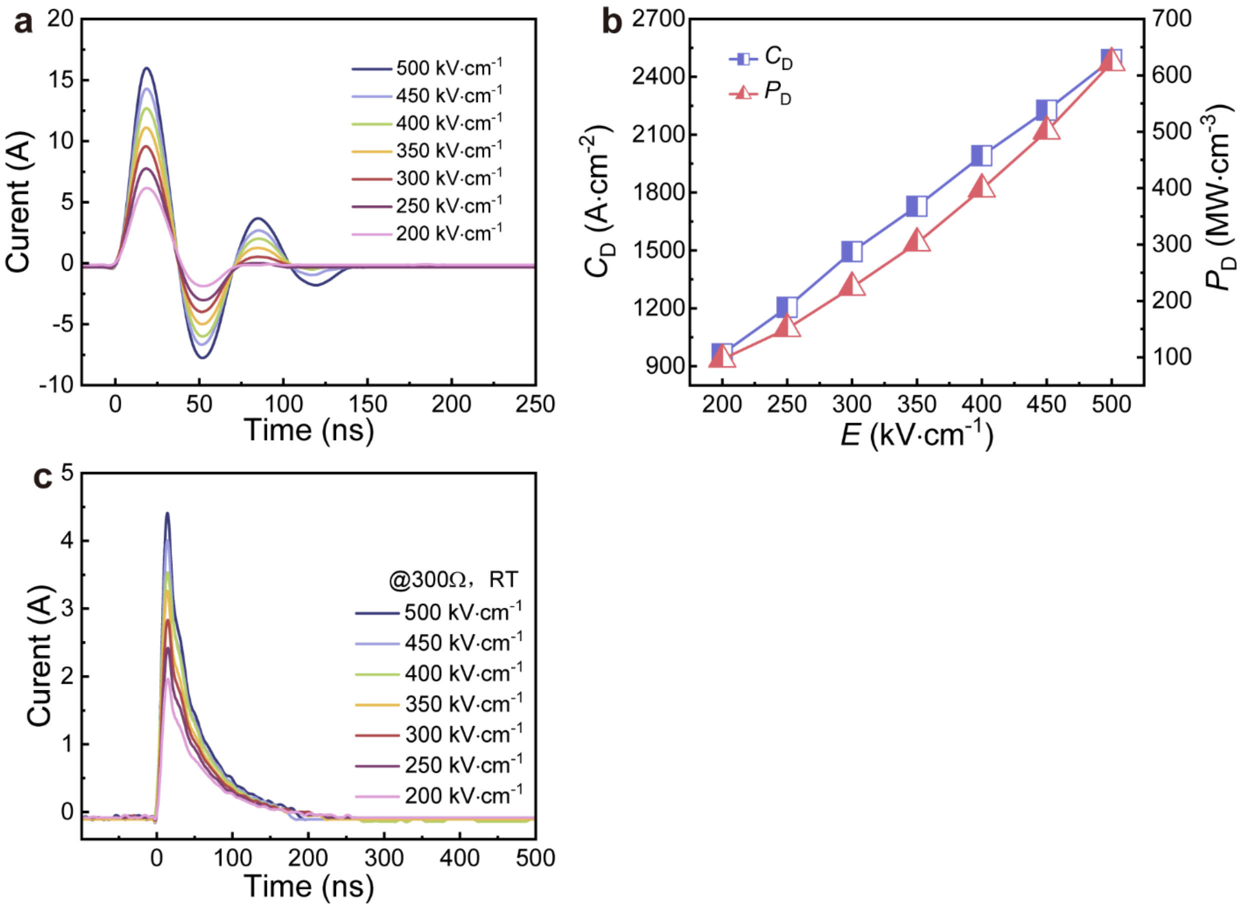

Figure S9. The charge/discharge performance of SBPLNN ceramics. a Underdamped discharge waveforms under different electric fields. **b** C_D and P_D values of SBPLNN as a function of electric field. **c** Overdamped discharge waveforms under different electric fields (R = 300 Ω).

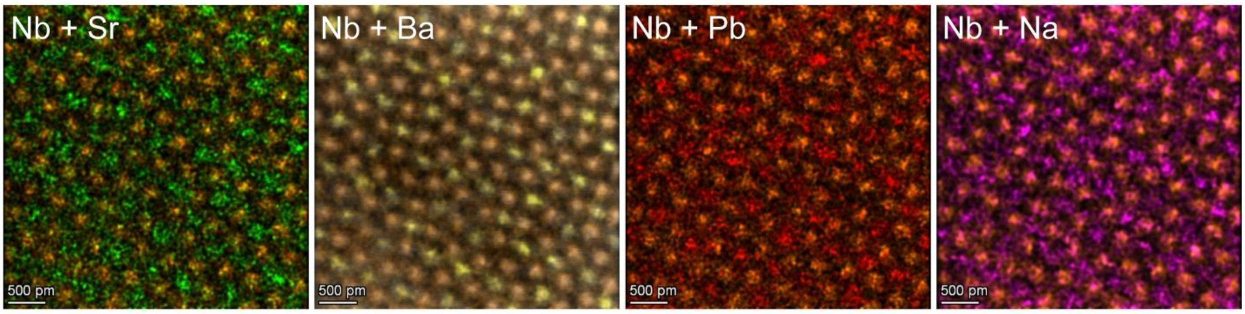

Figure S10. Atomic-scale element distribution of SBPLNN ceramics. The atomically resolved elemental mapping of Nb with Sr, Ba, Pb, and Na, respectively.

Table S1. Element distribution at A1 and A2 sites as calculated by EDS and the intensity of atom column.

Element	A1	A2	A1/A2
Total	33.53%	66.47%	0.5044
Sr	28.90%	71.10%	0.4065
Ba	34.47%	65.53%	0.5260
Pb	29.27%	70.73%	0.4138
La	35.72%	64.28%	0.5557
Na (estimated value)	39.29%	60.71%	0.6472

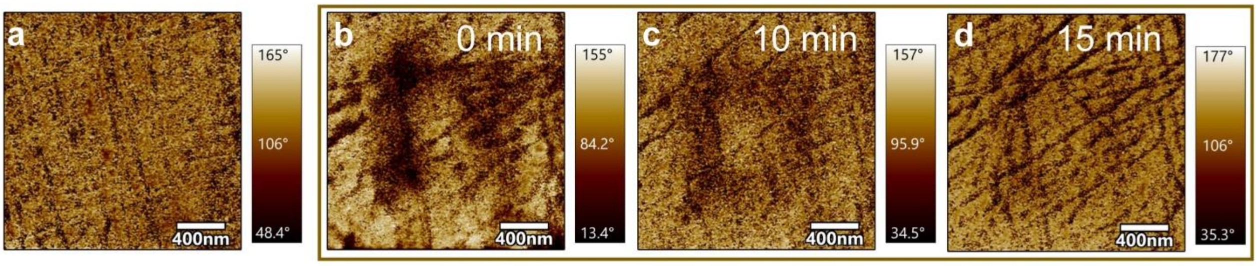

Figure S11. Domain structure and domain mobility of SBPLNN ceramics tested by PFM. a Out-of-plane PFM phase images for SBPLNN ceramics before poling, and **b-d** corresponding phase images after poling with a voltage of 40 V at different relaxation durations.

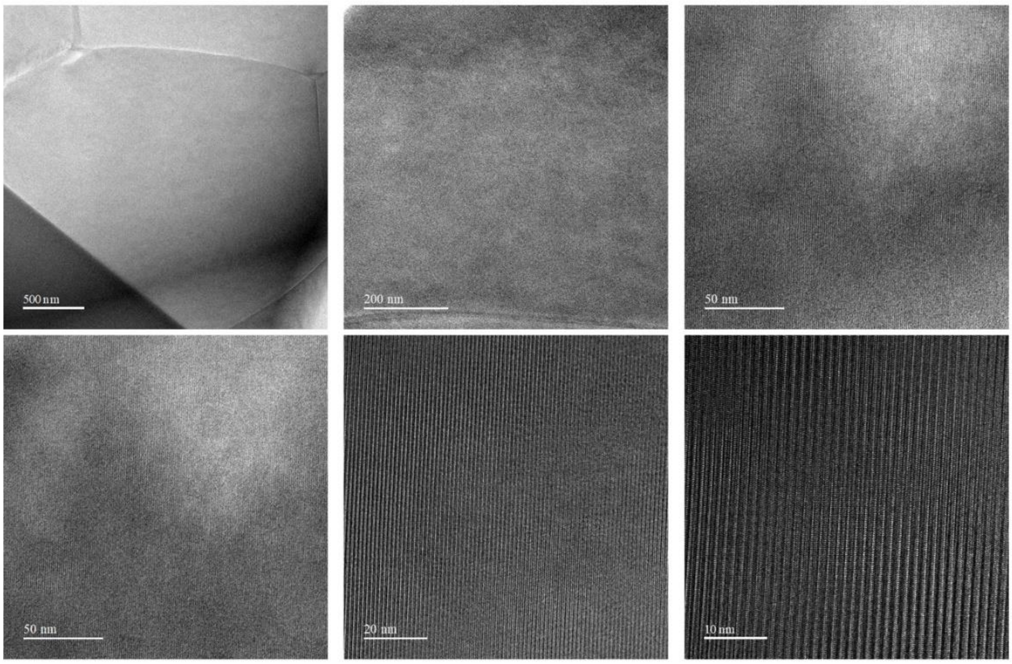

Figure S12. STEM of SBPLNN ceramics at different magnifications

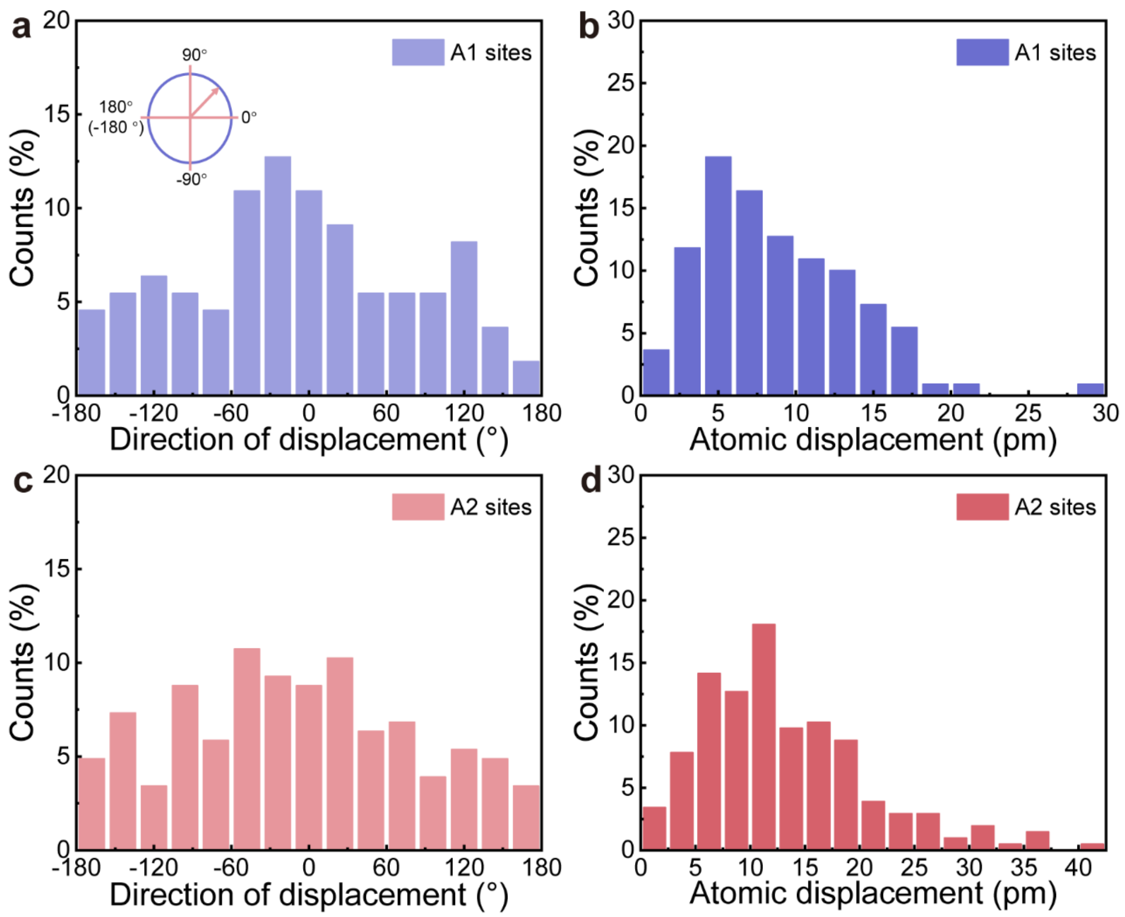

Figure S13. Lattice distortion in the high-entropy ceramics. The Statistical distribution bar chart of direction and magnitude of atomic displacement of **a-b** A1 and **c-d** A2 sublattice positions in SBPLNN ceramics.

Table S2. A comparison of resistance between SBPLNN high entropy ceramics and other classical TTBs ceramics.

Compositions	Resistance (kΩ)	test temperature (℃)	Ref.
**$(Sr_{0.2}Ba_{0.2}Pb_{0.2}La_{0.2}Na_{0.2})Nb_2O_6$**	**~ 250**	**500**	**This work**
$Sr_{0.7}Ba_{0.3}Nb_2O_6$	~ 150	500	[1]
$Sr_{0.5}Ba_{0.5}Nb_2O_6$	~ 100	500	[2]
$Sr_2KNb_5O_{15}$	~ 50	500	[3]
$Sr_2NaNb_5O_{15}$	~ 100	500	[4]

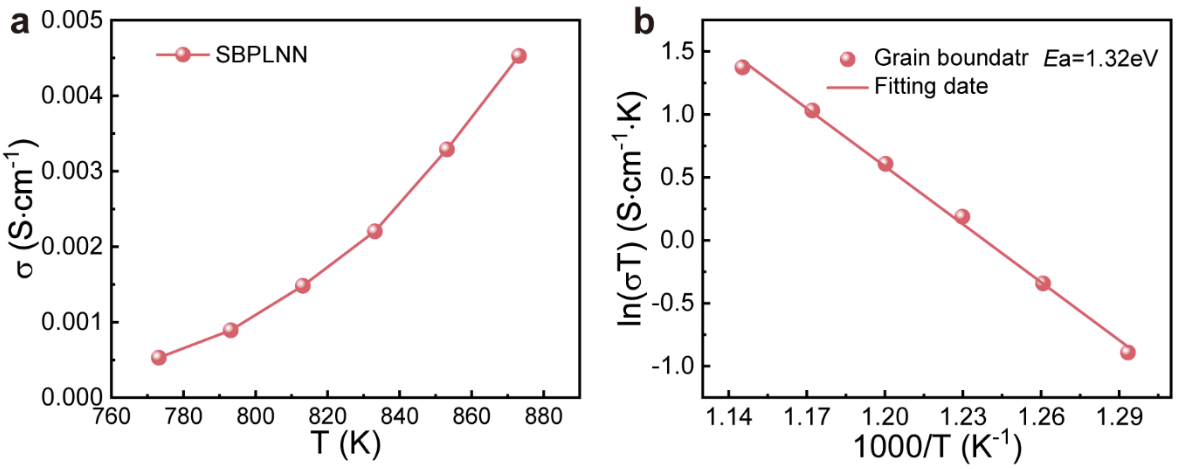

Figure S14. The insulating property of SBPLNN ceramics. a Calculated conductivity based on Zview fitted impedance data. **b** Arrhenius-type plots of resistivity as a function of 1000/*T*.

Table S3. A comparison of leakage current between SBPLNN high entropy ceramics and other reported TTBs and perovskite structure ceramics.

Compositions	Leakage current ($A\cdot cm^{-2}$)	Electric field ($kV\cdot cm^{-1}$)	Ref.
**$(Sr_{0.2}Ba_{0.2}Pb_{0.2}La_{0.2}Na_{0.2})Nb_2O_6$**	**$\sim 9.7\times10^{-8}$**	**300**	**This work**
**$(Sr_{0.2}Ba_{0.2}Pb_{0.2}La_{0.2}Na_{0.2})Nb_2O_6$**	**$\sim 5.7\times10^{-8}$**	**150**	**This work**
**$(Sr_{0.2}Ba_{0.2}Pb_{0.2}La_{0.2}Na_{0.2})Nb_2O_6$**	**$\sim 2.9\times10^{-8}$**	**50**	**This work**
$Gd_{0.03}Ba_{0.47}Sr_{0.455}Sm_{0.02}Nb_2O_6$	$\sim 5.0\times10^{-4}$	350	5
$Sr_{0.515}Ba_{0.47}Gd_{0.01}Nb_{1.85}Ta_{0.1}Sb_{0.05}O_6$	$\sim 1.2\times10^{-6}$	150	6
$Sr_{0.5}Ba_{0.47}Gd_{0.02}Nb_{1.8}Ta_{0.2}O_6$	$\sim 1.0\times10^{-6}$	150	7
$Sr_{0.53}Ba_{0.47}$ Nb_2O_6	$\sim 1.0\times10^{-5}$	150	7
$Sr_{1.82}Gd_{0.12}NaNb_5O_{15}$	$\sim 7.0\times10^{-7}$	150	8
$0.7Bi_{1.05}Fe_{0.99}Sb_{0.1}O_3$-$0.3BaTiO_3$	$\sim 2.0\times10^{-6}$	45	9
$BaTiO_3$@20 wt%SiO_2	$\sim 2.0\times10^{-8}$	50	10
$BaTiO_3$	$\sim 2.0\times10^{-6}$	50	10
$0.775Na_{0.5}Bi_{0.5}TiO_3$-$0.225BaSnO_3$+5wt.% MgO	$\sim 5.0\times10^{-7}$	90	11
$0.75Ba_{0.85}Ca_{0.15}Zr_{0.1}Ti_{0.9}O_3$-$0.25Bi(Mg_{0.5}Hf_{0.5})O_3$	$\sim 2.5\times10^{-8}$	120	12
$Sr_{0.7}Bi_{0.2}Ca_{0.1}TiO_3$	$\sim 5.0\times10^{-7}$	170	13
$Sr_{0.35}Bi_{0.35}K_{0.25}TiO_3$@2 wt.%$Er_2O_3$	$\sim 3.2\times10^{-7}$	100	14
$0.9(Na_{0.4}Bi_{0.4}Ba_{0.06}Sr_{0.14}Ti0.99Ta0.01O_3)$-$0.1NaNbO_3$	$\sim 7.0\times10^{-7}$	150	15
$0.8(0.67BiFeO_3$–$0.33BaTiO_3)$–$0.2Sr_{0.7}Bi_{0.2}Ca_{0.1}TiO_3$	$\sim 6.5\times10^{-8}$	80	16
$Ba_{0.95}Li_{0.05}TiO_3$	$\sim 3.0\times10^{-8}$	40	17

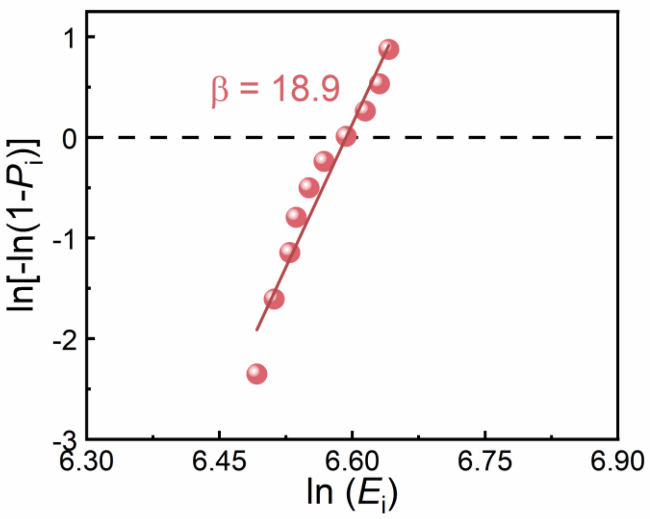

Figure S15. Weibull distributions of breakdown electric field for SBPLNN ceramics, the Weibull modulus β is determined as 18.9.

Reference

1. Luo C, *et al.* Promoting Energy Storage Performance of $Sr_{0.7}Ba_{0.3}Nb_2O_6$ Tetragonal Tungsten Bronze Ceramic by a Two-Step Sintering Technique. *ACS Appl. Energy Mater.* **4**, 452-460, (2021).
2. Peng H, *et al.* Superior Energy Density Achieved in Unfilled Tungsten Bronze Ferroelectrics via Multiscale Regulation Strategy. *Adv. Sci.* **10**, 2300227, (2023).
3. Wang H, *et al.* Pb/Bi-free Tungsten Bronze-Based Relaxor Ferroelectric Ceramics with Remarkable Energy Storage Performance. *ACS Appl. Energy Mater.* **4**, 9066-9076, (2021).
4. Zhang X, *et al.* Simultaneously Realizing Superior Energy Storage Properties and Outstanding Charge-Discharge Performances in Tungsten Bronze-Based Ceramic for Capacitor Applications. *Inorg. Chem.* **60**, 6559-6568, (2021).
5. Gao Y, *et al.* Ultrahigh Energy Storage in Tungsten Bronze Dielectric Ceramics Through a Weakly Coupled Relaxor Design. *Adv. Mater.* **36**, 2310559, (2023).
6. Yang B, *et al.* Enhancing Comprehensive Energy Storage Properties in Tungsten Bronze $Sr_{0.53}Ba_{0.47}Nb_2O_6$-Based Lead-free Ceramics by B-Site Doping and Relaxor Tuning. *ACS Appl. Mater. Interfaces* **14**, 34855-34866, (2022).
7. Yang B, *et al.* Remarkable energy storage performances of tungsten bronze $Sr_{0.53}Ba_{0.47}Nb_2O_6$-based lead-free relaxor ferroelectric for high-temperature capacitors application. *Energy Stor. Mater.* **55**, 763-772, (2023).
8. Cao L, *et al.* Ferroelectric-Relaxor Crossover and Energy Storage Properties in $Sr_2NaNb_5O_{15}$-Based Tungsten Bronze Ceramics. *ACS Appl. Mater. Interfaces* **14**, 9318-9329, (2022).
9. Li Y, *et al.* Relaxation degree and defect dipoles-controlled resistivity and leakage current in BF–BT-based ceramics. *J. Am. Ceram. Soc.* **106**, 2393-2406, (2022).
10. Xu C, *et al.* Tuning the microstructure of $BaTiO_3$@SiO_2 core-shell nanoparticles for high energy storage composite ceramics. *J. Alloys Compd.* **784**, 173-181, (2019).
11. Zhang L, *et al.* Antiferroelectric-like properties in MgO-modified $0.775Na_{0.5}Bi_{0.5}TiO_3$-$0.225BaSnO_3$ ceramics for high power energy storage. *J. Eur. Ceram. Soc.* **38**, 5388-5395, (2018).
12. Zhang X, *et al.* Achieved excellent energy storage properties and ultrahigh power density of $Ba_{0.85}Ca_{0.15}Zr_{0.1}Ti_{0.9}O_3$ lead-free ceramics modified by $Bi(Mg_{0.5}Hf_{0.5})O_3$. *J. Alloys Compd.* **968**, 172171, (2023).
13. Zhao P, *et al.* Novel Ca doped $Sr_{0.7}Bi_{0.2}TiO_3$ lead-free relaxor ferroelectrics with high energy density and efficiency. *J. Eur. Ceram. Soc.* **40**, 1938-1946, (2020).
14. Zhao P, *et al.* Improved dielectric breakdown strength and energy storage properties in Er_2O_3 modified $Sr_{0.35}Bi_{0.35}K_{0.25}TiO_3$. *Chem. Eng. J.* **403**, 126290, (2021).
15. Jiang Z, *et al.* Enhanced breakdown strength and energy storage density of lead-free $Bi_{0.5}Na_{0.5}TiO_3$-based ceramic by reducing the oxygen vacancy concentration. *Chem. Eng. J.* **414**, 128921, (2021).
16. Liu Z, *et al.* Ultrahigh Polarization Response along Large Energy Storage Properties in $BiFeO_3$–$BaTiO_3$-Based Relaxor Ferroelectric Ceramics under Low Electric Field. *ACS Appl. Mater. Interfaces* **14**, 53690-53701, (2022).
17. Sarraf S, *et al.* Enhanced electrocaloric response and energy storage performance of Li-substituted $BaTiO_3$ ceramics. *J. Am. Ceram. Soc.* **105**, 6196-6206, (2022).
